# The College of Nursing and Its Opponents: A Remarkable Volte Face

**Published:** 1917-12-01

**Authors:** 


### THE MATRONS' AND SISTERS' DEPARTMENT.

### THE COLLEGE OF NURSING AND ITS OPPONENTS.

### A Remarkable Volte Face.

THE College of Nursing has progressed steadily and increasingly from the commencement. Its Register is growing in numbers and importance. The devoted service of the British Women's Hospital Committee, under Lady Cowdray's direction, in raising the Nation's Fund for Nurses as a thank-offering from the British Empire to British Nurses, and its reception by the people of all classes in these islands, should speedily place behind the College an adequate Endowment and Building Fund. They have also in hand the Tribute Fund, to enable the College to make provision for those of its members who, through no fault of their own, are in need or in suffering, thus rendering back to the nurses themselves some small part of the immeasurable debt owed to them by the community to which they have ministered so devotedly. All honour, then, to Lady Cowdray and the British Women's Hospital Committee, who, by raising these eleemosynary gifts from a grateful Nation and Empire, will provide a just recognition of splendid services, on the same basis as other professions have successfully appealed for funds for the building and endowment of their colleges.

The College of Nursing is designed in the first instance to stand in the same relation to the profession of nursing as the Colleges of Physicians and Surgeons have to the professions of medicine and surgery. To fulfil its mission the College requires of necessity an Endowment Fund, with the means to enable it to offer every inducement and facility to those who show special aptitude for the higher branches of nursing, including the provision of scholarships as the accepted form of enabling successful candidates to pursue special courses of study, and to travel, with a view to acquainting themselves with the methods which govern nursing practice in other countries. The under-payment of the British nurse has long been a scandal. Although steps are being and have been taken in recent years to improve matters in this respect, much still remains to be done. This it will be the privilege of the College of Nursing to further and secure by making the inequalities known, and producing the necessary evidence to secure a just remedy with completeness and promptitude. The College of Nursing will further establish standardisation and organisation throughout the whole nursing profession. The absence of both has long been realised, and the need for them is universally felt by members of the nursing profession, by the nurse-training schools, and by all who have the best interests of British nursing at heart.

A knowledge of all these circumstances has secured widespread support for the College of Nursing, which has justly been welcomed by the British Press of all shades of opinion. Nothing succeeds like success, and if any member of the nursing profession still doubts the success and steady progress to fulfilment of the College of Nursing, she has only to read carefully the proceedings at the meeting at the College of Ambulance, Vere Street, on the afternoon of Saturday, November 17, and to couple with them the fact that the opponents' leader in her paper publishes with approbation statements that the mention of the British Women's Hospital Committee was received (1) "with hisses," and (2) "the hissing "was the most significant and encouraging sign "of the awakening professional conscience amongst "trained nurses." Now we have taken pains to ascertain exactly what did occur. There were upwards of 200 people present, and the reporter's statement is: "There was one energetic issue of steam in an isolated corner, momentary and almost ashamed," and well it might be. This may be taken as evidence that the overwhelming majority of the nurses present took no part whatever in this shameful exhibition of party exuberance. A full *résumé* of Miss Brodrick's address appeared in *The Nursing Mirror*, but the opponents published as they describe what "Miss Brodrick said in part."

The opponents of the College, as voiced by their leader, whilst confessing that the object of the College of Nursing is to organise the profession, have denounced the raising of funds for its buildings, colleges, endowment, and scholarships through the British Women's Hospital Committee as **"a determined endeavour to pauperise the profession,"** adding, **"there can be no freedom for a profession organised on charity."** Further, the College **"has no right to seek a huge sum of money from the public, for such a system must deprive nurses of economic and professional independence."** It is **"demoralising patronage by wealthy, leisured women." "The ignorance of so many hospital matrons of political economy, and their reactionary attitude towards their supporters, are greatly to blame for the humiliating position in which the nursing profession is now being placed."** Finally, with an inconsistency which never fails the leader of the opponents of the College, they stated so recently as April last that, an educational endowment would be **"an undoubted benefit to the nursing profession, and indeed we have publicly pleaded for it for the last twenty years."**

THE HOSPITAL has continuously exposed the absurdity of the attempts thus made to prejudice the nurses and to set back the progress of the nursing profession. With a view to stop these attempts, to render them impotent for the future, and in justice to the British Press, which has been denounced for helping the cause of the nurses as "a shameful exhibition of tyranny and a disgrace to the British Press," we feel it to be a privilege and duty to expose the humbug and hollowness on which these attacks on the College and the British Women's Hospital Committee, in fact, rest. This will be convincingly demonstrated by the publication of Mrs. Bedford Fenwick's declared opinions on the questions at issue.

**An Address given at an International Congress of Nurses in America in 1901 by Mrs. Bedford Fenwick.**

**The following are Verbatim Extracts:—**

"But educational advantages for nurses mean a "direct gain to the public, and I think you will "agree with me that **it is not just that the whole "financial burden of the further advance of nurs- "ing should be entirely borne by nurses them- "selves. In other and richer professions the public "take their share in financial support. Witness "the magnificent universities, the endowed profes- "sional chairs, the medical colleges, public libraries, "and numerous organisations which afford oppor- "tunities of study to different sections of workers, "resulting in the ultimate benefit of the community "at large, but owing their existence to the munifi- "cence of a comparatively few public-spirited "persons.**

"I claim that the time has come when nurses "need their educational centres, their endowed "colleges, their chairs of nursing, their "university degrees, and State registration, "and the present seems the psychological "moment to come to the public, not as "strangers, but as professional workers known "and trusted through the length and breadth of "the land, and to urge that, as nurses pour out "on its behalf a skill and devotion for which gold "is no real recompense, **the public shall now prove "its appreciation and interest in the noble work "of nursing by giving something of its wealth to "place nursing education and the status of the "trained nurse on a strong financial basis.**

**"Is it too much to hope that the wealthy will "come forward and found colleges of nursing— "colleges in which the teaching power of the "profession would be focussed and centred, which "would put the apex on our training course, and "by improving the standard of nursing the sick "confer a real and lasting benefit on humanity at "large?"**

It only remains for us to tender the grateful thanks of British people and Nurses to the proprietors and editors of the British Press who have already given such generous support to the College and the British Women's Hospital Committee. It rests with each British nurse to defend her best interests by making it her business to place her name on the College Register quickly, that she may vote at the first election of members to the College Council next spring.

